# *Astragalus mongholicus (Fisch.) Bge* Improves Peripheral Treg Cell Immunity Imbalance in the Children With Viral Myocarditis by Reducing the Levels of miR-146b and miR-155

**DOI:** 10.3389/fped.2018.00139

**Published:** 2018-06-21

**Authors:** Zhen Zhang, Xinlun Dai, Ji Qi, Yu Ao, Chunfeng Yang, Yumei Li

**Affiliations:** ^1^Department of PICU, The First Hospital of Jilin University, Changchun, China; ^2^Clinical Medical College, Jilin University, Changchun, China

**Keywords:** miR-146b, miR-155, Treg cell immunity, viral myocarditis, cytokine

## Abstract

Viral myocarditis (VMC) is a common cardiac disease, however, there still lacks an effective therapeutic strategy for VMC. *Astragalus mongholicus (Fisch.) Bge* (AB), a Chinese herb with some functional metabolites, may have some pharmacological effects on VMC. AB ingredients were measured by a full-scan LCQ mass spectrum. We aimed to explore the effects of AB on the VMC children by investigating peripheral Treg cell homeostasis. A total of 68 VMC children were random and evenly assigned into an AG group (received 10-mL AB oral liquid daily), and a CG group (received placebo daily). Peripheral blood mononuclear cells (PBMC) were obtained from peripheral blood and Treg cells were isolated. The levels of miR-146b, miR-155, Treg immunity activity and myocarditis biomarkers were measured in Treg cells. There were four main components (sucrose, calycosin, Astragaloside IV and calycosin-7-glucoside) in AB. The cases sinus tachycardia, frequent premature ventricular contractions, and supraventricular tachycardia were significantly reduced in the AG group (*P* < 0.05). Meanwhile, the myocardial enzymes and cardiac function indexes were improved in the AG group when compared with the CG group (*P* < 0.05). The time of electrocardiogram recovery, symptom duration and hospital stay was shorter in the AG group than in the CG group (*P* < 0.05). The levels of miR-146b and miR-155 were higher in the CG group than in the AG group (*P* < 0.05). The levels of ROR-γt (retinoic acid receptor-related orphan nuclear receptor gamma), FoxP3 (forkhead transcription factor), IL-10 (interleukin-11) and TGF-β (transforming growth factor beta) were lower in the CG group than in the AG group (*P* < 0.05). In contrast, the levels of IL-17, IL-21, CK-MB (creatine kinase-MB), cTnI (cardiac troponin I), GrB (granzyme B), sFasL (soluble fas ligand) and caspase-3 were higher in the CG group than in the AG group (*P* < 0.05). Furthermore, the levels of ROR-γt, FoxP3, IL-10, and TGF-β were positively, whereas the levels of IL-17, IL-21, CK-MB, cTnI, GrB, sFasL and caspase-3 were negatively, associated with the levels of miR-146b and miR-155 (*P* < 0.05). AB treatment improved cardiac functions, peripheral Treg cell immunity imbalance in the children with VMC by reducing the levels of miR-146b and miR-155.

## Introduction

Viral myocarditis (VMC) is caused by enterovirus and adenovirus infection, and the incidence shows a rising trend recently. *Astragalus mongholicus (Fisch.) Bge* (AB) is one of the most important traditional Chinese herbs and has some functional metabolites. AB has been reported to prevent and treat inflammation by inhibiting lipopolysaccharide-induced production of TNF-α and IL-8, and suppressing the p38 signaling pathway (1). Other work also showed the anti-inflammatory properties of AB (2). AB polysaccharides can significantly improve serum and liver antioxidant activities and reduce peroxidative lipid levels (3). On the other hand, antioxidant and anti-inflammatory treatment is often considered for VMC therapy (4–6). Thus, AB may have beneficial and pharmacological effects on VMC. However, the related molecular mechanism remains unknown.

VMC can cause myocardial injury by disturbing autoimmunity balance. Human CD4^+^ T regulatory cells (Tregs) are phenotypically and functionally cells regulating inflammatory and autoimmune activities, and T helper (Th17) cells are correlated with the pathogenesis of autoimmune diseases. Interleukin (IL)-17-producing Th17 cells participate in the pathogenesis of myocarditis. In the animal model with autoimmune myocarditis, the expression of Th17-specific transcription factor, retinoic acid receptor-related orphan nuclear receptor gamma (ROR-γt), was increased (7). FoxP3 (forkhead transcription FoxP3-expressing natural Treg, including CD25^+^ and CD25^−^ cells, plays an important role in inhibiting various autoimmune diseases, and depletion of these cells, which will result in autoimmune diseases (8).

Activation of TGF-β (transforming growth factor beta) regulates myofibroblast differentiation and myocardial fibrosis development (9). According to an earlier report, cTnT (cardiac troponin T) and CK-MB (creatine kinase-MB) are sensitive biomarkers for the diagnostic evaluation of myocarditis (10). High-level cTnI (cardiac troponin I) has been reported to be associated with the clinical and echocardiographic evidence of severe myocarditis (11). Serum GrB (granzyme B) and sFasL (serum soluble fas ligand) have also been found in the children with VMC (12). IL-10 produced by T cells, can control *Trypanosoma cruzi* and protect against acute myocarditis (13). Additionally, the increase in plasma levels of cTnI and IL-17 has also been found in VMC patients (14). Active caspase-3 plays a critical role in apoptotic cardiomyocyte death in fatal myocarditis (15). On the other hand, changes of Th17/Treg, related cytokines and transcription factors, are also the main reasons in the pathogenesis of inflammatory diseases (16).

miRNAs are small non-coding RNAs and the changes in the levels of miR-1 have been observed in arrhythmia and heart disease (17). miR-155 is also a potential therapeutic target for treating VMC (18). Furthermore, miR-146b (19) and miR-155 (20) are two important miRNAs involving in the regulation of immune response. miR-146b and miR-155 may affect Treg immunity activity and myocarditis biomarkers in the peripheral blood of VMC children. AB may have some pharmacological effects on VMC. We explored the effects of AB on peripheral Treg cell immunity imbalance in VMC children, and analyzed the relationship between the expression of miR-146b and miR-155, and Treg immunity activity and myocarditis biomarkers in the peripheral blood of VMC children.

## Materials and methods

### Analysis of AB ingredients

A total of 500-g AB were purchased from Affiliated Hospital to Changchun University of Chinese Medicine, and identified by Professor Bo Liu. Two liters of water was added to AB and boiling water for twice, each 1 h. The liquid was mixed and filtered by using 0.22-μm Whatman filter paper (Millipore, Bedford, MA, USA). The filtrate was concentrated to about 1 liter, and centrifuged at 12,000 g for 30 min. Finally, the liquid was sterilized at 121°C for 30 min. 2-mL AB was added to methanol to make final 20 mL totals.

The standard calycosin-7-glucoside was purchased from the National Institute for the Pharmaceutical and Biological Products of China (Beijing, China). Sucrose, calycosin, and astragaloside IV were purchased from Sigma (St. Louis, MO, USA). The main components of AB were determined by using a Finnigan LCQ^DECA^ mass spectrometer (Finnigan, San Jose, CA, USA). The LC system concluded a P4000 pump, a 6000LP photodiode array detector, and an AS 3000 autosampler (TSP, San Jose, CA, USA). Chromatography-mass spectrometry conditions included Lnertsil ODS-c18 column (4.6 × 250 mm, 5 μm); The main ingredients were eluted by acetonitrile–water gradients as follows: 0–20 min, 5–30% acetonitrile; 21–40 min, 31–40% acetonitrile; and 41–60 min, 41–100% acetonitrile; Column temperature, 25°C; Injection volume, 20 μL; Chromatogram wavelength, from 190 to 400 nm. The results showed there was good separation at 225 nm. Therefore, 225 nm was used as the detection wavelength. Mass Spectrometry were designed as follows: ESP voltage, 2–3.5 kV; sheath gas (N2), 80 au; auxiliary gas (N2) pressure, 15 AU; collision gas, helium; capillary temperature, 350°C; capillary voltage, 5 V; All ion fragmentation mode, 50~2,000 mA; MSn collision energy: 40%.

### Participants

Before the present experiment, all processes were approved by the Human Research Ethical Committee of The First Hospital of Jilin University. VMC was confirmed in routine autopsy specimens by immunofluorescent techniques according to an earlier report (21). Furthermore, VMC refers to the virus invasion of the heart caused by focal or diffuse myocardial interstitial inflammatory changes and myocardial fibrosis or necrotic lesions. The pathogenesis is complex and virus infection can cause an autoimmune response. Pathological responses can be involved in myocardium and interstitial changes, which can be pathological basis of electrocardiography (ECG) (22). The patients with VMC often have ECG abnormalities (23). In order to understand the characteristics of ECG changes in VMC children, all patients were confirmed by using ECG analysis.

### Inclusion criteria

Children included in this study were clinically diagnosed as VMC, and met the following criteria: (1) the first incidence of VMC; (2) did not take any drug after the onset of disease; (3) Virus was isolated from children's blood or virus nucleic acid could be determined; (4) IgM antibody showed positive responses; (5) the serum had the same type of antibody titer; (6) The guardian of children voluntarily signed an informed consent.

### Exclusion criteria

The patients were excluded if they had the following situation: (1) heart failure and severe arrhythmia; (2) past history of congenital heart disease, rheumatic myocarditis, endocardial inflammatory inflammation and toxic myocarditis, or serious heart, liver kidney, brain and other important organ diseases and allergic asthma; (3) self-medication could have an impact on the test results; (4) they did not accept follow-up.

### Patients grouping

After the selection of inclusion and exclusion criteria, a total of 68 VMC patients were selected in the First Hospital of Jilin University from March 2015 to May 2016, all of whom met the diagnostic criteria of VMC, and did not receive antiviral drugs and other medical treatment. All patients were evenly assigned into two AG (the children received 10-mg AB oral liquid daily) and CG (the children received placebo daily) groups. There was no significant difference in clinical data between two groups of children (*P* > 0.05).

### Clinical evaluation criteria

The treatment effect was evaluated by using the following clinical criteria: (1) markedly effective, the clinical manifestations were completely disappeared; ECG and laboratory tests were normal; chest X-ray showed that the ratio of cardiac dimensions to chest dimensions were about 50%; (2) effective, clinical manifestations were improved; ECG and laboratory results were improved or returned to normal; the chest X-ray showed that the ratio of cardiac dimensions to chest dimensions were more than 50%; (3) ineffective, clinical manifestations were obvious. Laboratory and ECG tests did not change significantly.

Two-mL peripheral blood was collected from VMC children. Serum was obtained via centrifugation at 1,500 g at 4°C for 10 min. Serum activities of CK (creatinine kinase) and LDH (lactate dehydrogenase) were measured by using the kits (Cat. No. ab155901 and ab102526) from Abcam Trading (Shanghai) Company Ltd. (Shanghai, China). Serum activity of CK-MB (creatinine kinase MB) was measured by using the kit (Cat. No. PRB-5047) from Cell Biolabs, Inc. (San Diego, CA, USA). CO (cardiac output) was measured by using thermodilution according to an earlier report (24). EF (ejection fraction) was detected by using a noninvasive method (25). SV (stroke volume) was examined by using a modified method (26).

### Treg cells isolation

Five-mL peripheral blood was collected from VMC children. Peripheral blood mononuclear cells (PBMC) were isolated from 2-mL blood by using Ficoll separation and density gradient centrifugation. Treg cells were further isolated from PBMC of all subjects by using a CD4^+^CD25^+^Treg cell isolation kit (Miltenyi Biotec Technology and Trading (Shanghai) Co., Ltd., Shanghai, China) according to manufacturer's instruction.

### Real-time quantitative polymerase chain reaction (qPCR) analysis of the relative levels of miR-146b and miR-155 in treg cells

miRNeasy Serum/Plasma kit and one step primer script miRNA cDNA synthesis kit (Qiangen, Valencia, USA) were used to isolate miRNA from Treg cells and synthesize cDNA. cDNA samples were measured by using fluorescence qPCR. RNU6B was used as an internal control for normalizing relative levels of miR-146b (forward primer, 5′-cctggcactgagaactgaat-3′; reverse primer, 5′-gggcaccagaactgagtcc-3′) and miR-155 (forward primer, 5′-ctgttaatgctaatcgtgat-3′; reverse primer, 5′-ttaatgctaatatgtaggg-3′).

### qRT-PCR analysis of relative mRNA levels of FoxP3 and ROR-γt in treg cells

Relative levels of FoxP3 (forward primer, 5′-atgcccaaccccaggcctgg-3′; reverse primer, 5′-ccctggaaggttccccctg-3′) and ROR-γt (forward primer, 5′-CAGCAGCGGCAACAGCAGCAAC-3′; reverse primer, 5′-GGCCCAGACCTGAGGCTTTC-3′) were measured by using fluorescent quantitative PCR by using β-actin (forward primer, 5′-tgcgtgacattaaggagaag-3′; reverse primer, 5′-ccattggcaatgagcggttc-3′) as an internal control.

### Measurement of cytokines and myocardial injury indicators

Whole protein was isolated from Treg cells by using a protein isolation kit (Miltenyi Biotec Technology and Trading (Shanghai) Co., Ltd., Shanghai, China) in accordance with manufacturer's instruction. ELISA kits for IL-10 (Cat No. ab46034), TGF-β (Cat No. ab46034), IL-17(Cat No. ab83688), IL-21(Cat No. ab119542), CK-MB (Cat No. ab193696), cTnI (Cat No. ab200016), GrB(granzyme B, Cat No. ab346142), sFasL (Cat No. ab100515) and caspase-3 (Cat No. ab181418) were used to measure their protein levels in cell lysis. All kits were purchased from Abcam Trading (Shanghai) Company Ltd. (Shanghai, China).

### Flow cytometry analysis of the level of FoxP3/RoRgt expression

Anti-human/mouse FITC–anti-CD4, PerCP5.5–anti-CD25, FoxP3-PE and ROR-γt -PE was purchased from eBioscience (San Diego, CA, USA) R&D Systems (Minneapolis, MN, USA). To confirm the level of Foxp3/ROR-γt expression, the Treg cells were also stained with FITC–anti-CD4, PerCP5.5–anti-CD25 and PE-anti-Foxp3 or RORγt antibodies (eBiosciences). The Cells were determined by using by flow cytometry (Accuri C6 flow cytometer; BD, USA), and data were analyzed using FlowJo 7.6.5 (Tree Star, Ashland, OR, USA).

### Statistical methods

All data were presented as mean values ± S.D. (standard deviation). Paired *T*-test was used to analyze the significant difference between two variables and Pearson test was used to analyze the correlation between two parameters. All data were analyzed by using SPSS20.0 software (Chicago, IL, USA). Statistical difference was significant if *P* < 0.05.

## Results

### AB ingredients

**Figure 1:**
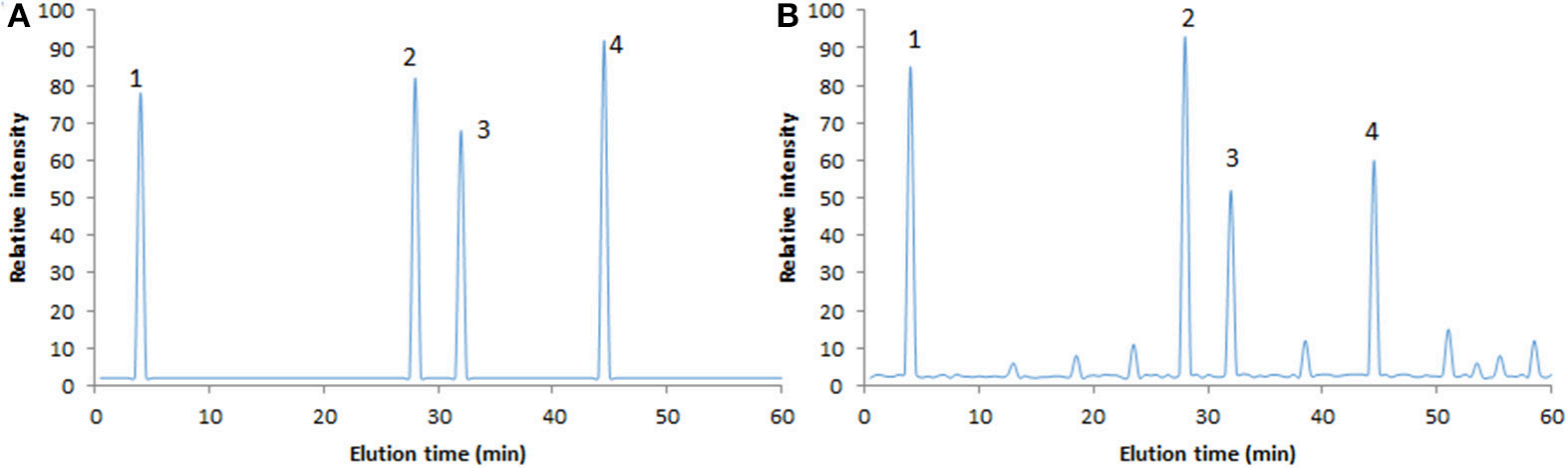
HPLC analysis of AB ingredients. **(A)** The standards of sucrose, calycosin, Astragaloside IV, and calycosin-7-glucoside. **(B)** The main ingredients of AB.

**Figure 2:**
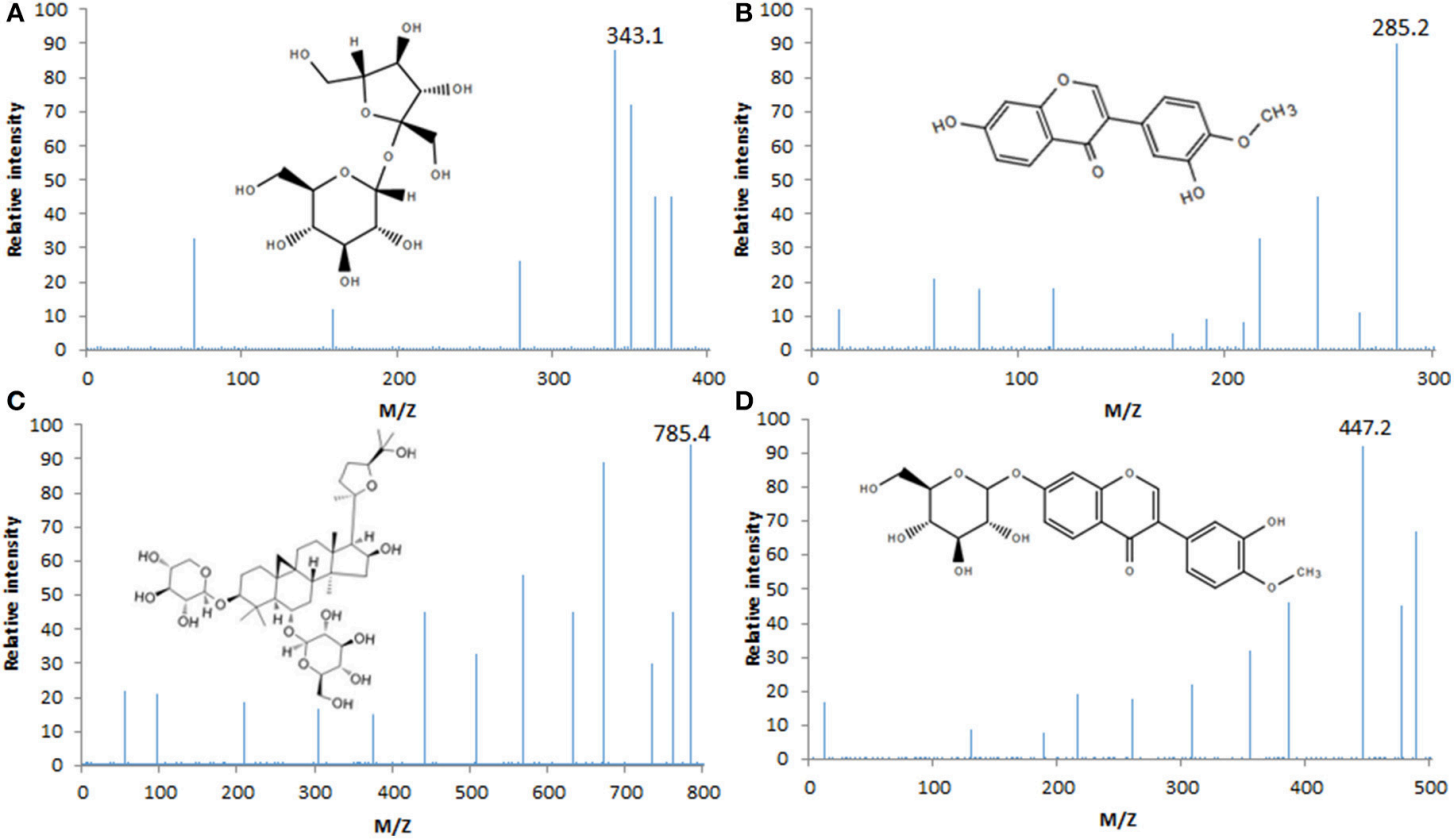
Analysis of LCQ mass spectrometer of AB ingredients. **(A)** The MW of sucrose was 343 in a [M+H]^+^ mode. **(B)** The MW of calycosin was 285 in a [M+H]^+^ mode. **(C)** The MW of Astragaloside IV was 785 in a [M+H]^+^ mode. **(D)** The MW of calycosin-7-glucoside was 447 in a [M+H]^+^ mode.

HPLC analysis showed that there were four main components (sucrose, calycosin, Astragaloside IV and calycosin-7-glucoside, Figure 1B) in AB according to the results of standards (Figure 1A). Analysis of LCQ mass spectrometer further proved that the main ingredients of AB were sucrose (MW, 343, Figure 2A), calycosin (MW, 285, Figure 2B), Astragaloside IV (MW, 785, Figure 2C) and calycosin-7-glucoside (MW, 447, Figure 2D) in a [M+H]^+^ mode.

### Clinical characteristics

In this study, the main clinical manifestations of palpitations, fatigue, weakness in front of heart, pale, dizziness, and ST-T segment abnormalities were similar between two groups. Myocardial enzyme test showed CK-MB (creatine kinase MB)> 50U/L. The statistical differences were insignificant for gender distribution, age, VMC duration, viral infection, VMC complications and other clinical characteristics (*P* > 0.05, Table 1).

**Table 1:** Comparison of clinical characteristics between the CG group and the AG group.

	**AG (*n* = 43)**	**CG (*n* = 43)**	***t*-value/χ^2^**	***P*-values**
Age, years	5.2–11.3	5.3–11.7	−0.58	0.29
Sex (male/female)	18/16	17/17	0.06	0.81
VMC duration, days	8.34 ± 3.36	8.07 ± 3.21	0.12	0.46
Viral infection			0.35	0.84
Intestinal tract	14	15		
Respiratory tract	18	16		
Other or no obvious infection	2	3		
VMC complications			0.40	0.94
Sinus tachycardia	14	12		
Frequent ventricular premature beats	10	11		
Supraventricular tachycardia	7	8		
Atrioventricular block above grade II	4	3		
Body mass index (kg/m^2^)	20.5 ± 3.4	21.1 ± 3.7	−0.91	0.20
TC (mg/dL)	3.62 ± 0.23	3.56 ± 0.26	0.66	0.26
TG(mg/dL)	1.43 ± 0.11	1.39 ± 0.13	0.54	0.18
HDL-C(mg/dL)	1.08 ± 0.09	1.10 ± 0.11	−0.69	0.37
LDL-C(mg/dL)	2.17 ± 0.14	2.06 ± 0.17	0.86	0.12
First-degree atrioventricular block, *n* (%)	3 (2.1)	0 (0)		
Second-degree atrioventricular block, *n* (%)	14 (9.6)	0 (0)		
Second-degree I type atrioventricular block, *n* (%)	6 (4.1)	0 (0)		
Second-degree II type atrioventricular block, *n* (%)	5 (3.4)	0 (0)		
Third-degree atrioventricular block, *n* (%)	4 (2.7)	0 (0)		
Atrioventricular block, *n* (%)	8 (5.5)	0 (0)		
Right bundle branch block, *n* (%)	3 (2.1)	0 (0)		
Left bundle branch block, *n* (%)	20 (13.7)	0 (0)		
Atrial fibrillation and atrial flutter, *n* (%)	2 (1.4)	0 (0)		
Short bursts of tachycardia, *n* (%)	11 (7.5)	0 (0)		
Ventricular premature beats, *n* (%)	50 (34.2)	0 (0)		
Abnormal waves, *n* (%)	2 (1.4)	0 (0)		
QRS low voltage, *n* (%)	3 (2.1)	0 (0)		
ST-T changes, *n* (%)	15 (10.3)	0 (0)		

### Comparison of therapeutic results

After treatment, AB treatment was more effective in the AG group than the CG group. Symptoms duration and hospital stay was shorter in the AG group than the CG group too (Table 2, *P* < 0.05). Before the therapy there was no significant difference for ECG results were better in the AG group than the CG group and recovery time was shorter in the AG group than the CG group (Table 2, *P* < 0.05).

**Table 2:** Therapeutic results between AG and CG groups.

**Parameters**	**AG**	**CG**	**χ2 or *t* values**	***P*-values**
**EFFECTIVE RESULTS**				
Remarkably effective	21	7	36.19	0.01^*^
Effective	22	5		
Ineffective	1	22		
Symptoms duration, days	6.01 ± 1.15	9.28 ± 2.63	4.78	0.01^*^
Hospital stay, days	8.35 ± 1.72	11.35 ± 3.02	5.13	0.01^*^
**ECG RESULTS**				
Sinus tachycardia	3	8	6.73	0.08
Frequent ventricular premature beat	2	5		
Supraventricular tachycardia	1	3		
ECG recovery time, days	10.43 ± 1.63	12.22 ± 2.65	11.70	0.02^*^

* *P < 0.05 vs. a control group*.

### Myocardial enzymes and cardiac function recovery

*P* < 0.05, and the improvement rate of the treatment group were significantly higher than the control group, *P* < 0.05, as shown in **Tables 4**, **5**; Both groups did not appear during the treatment of liver and kidney dysfunction, severe allergic reaction or hypotension and other serious adverse reactions.

Serum activities of CK, CK-AB, and LDH were significantly reduced in the AG group when compared with the CG group (Table 3, *P* < 0.05) after 14-day treatment. Meanwhile, CO, EF, and SV were significantly increased in the AG group when compared with the CG group (Table 4, *P* < 0.05) after 14-day treatment. All these results suggest that AB improves cardiac functions of VMC children. There were no liver and kidney dysfunction, severe allergic reaction and hypotension after the therapy.

**Table 3:** Comparison of myocardial enzyme between two groups before and after treatment.

		**CK (IU/L)**	**CK-MB (IU/L)**	**LDH (IU/L)**
*Astragalus mongholicus (Fisch.) Bge* group	Before therapy	218.00 ± 56.97	32.95 ± 10.56	285.08 ± 60.66
	After therapy	103.58 ± 40.64^*^^#^	12.28 ± 7.34^*^^#^	155.43 ± 46.67^*^^#^
Control group	Before therapy	221.65 ± 60.69	35.08 ± 11.59	278.88 ± 68.01
	After therapy	238.08 ± 44.59	32.07 ± 8.39	297.36 ± 51.32

*CK, creatinine kinase; CK-MB, creatinine kinase MB; LDH, lactate dehydrogenase*.

* *P < 0.05 vs. the group before treatment*.

# *P < 0.05 vs. control group*.

**Table 4:** Comparison of cardiac function between two groups before and after treatment.

		**CO/(L/min)**	**EF (%)**	**SV (ml)**
*Astragalus mongholicus (Fisch.) Bge* group	Before therapy	4.65 ± 0.80	52.94 ± 7.63	60.87 ± 7.66
	After therapy	6.72 ± 1.59^*^^#^	72.42 ± 9.44^*^^#^	79.45 ± 6.61^*^^#^
Control group	Before therapy	4.67 ± 0.72	55.08 ± 7.53	59.39 ± 9.85
	After therapy	4.83 ± 0.95	51.85 ± 8.47	58.36 ± 7.34

*CO, cardiac output; EF, ejection fraction; SV, stroke volume*.

* *P < 0.05 vs. the group before treatment*.

# *P < 0.05 vs. control group*.

### miR-146b and miR-155 expression levels

**Figure 3:**
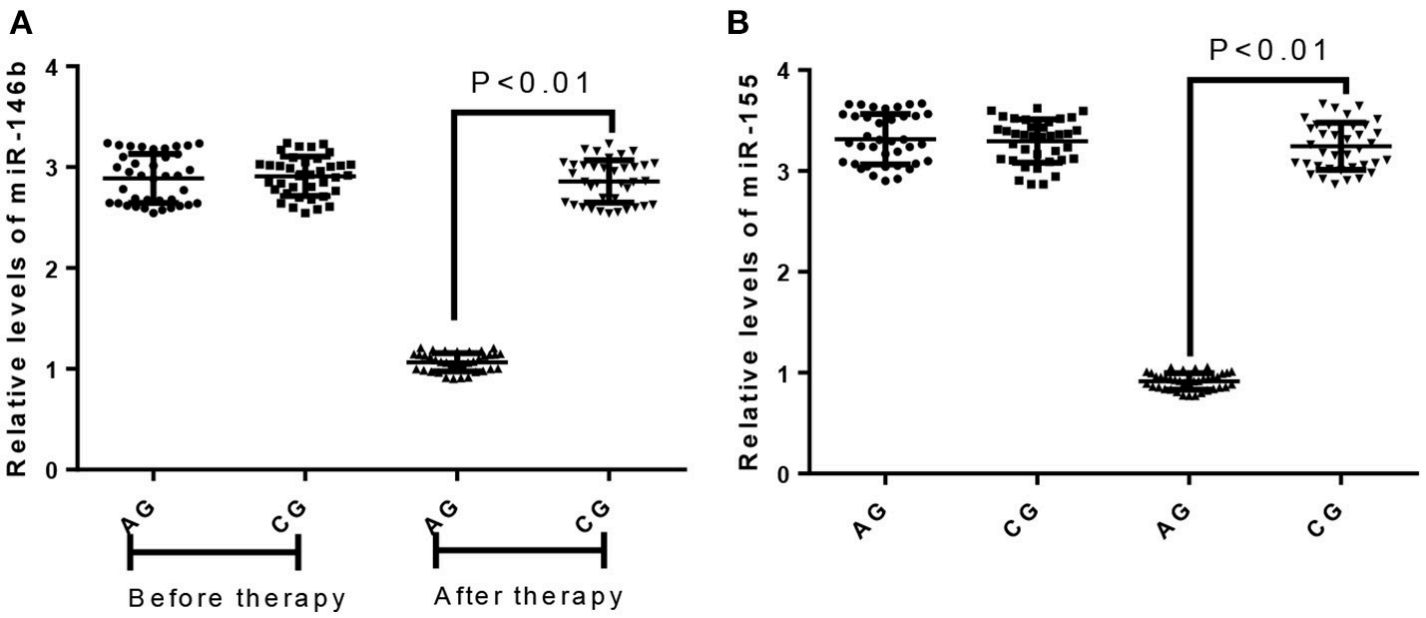
The effects of AB treatment on relative levels of miR-146b and miR-155. **(A)** The effects of AB treatment on relative levels of miR-146b between AG and CG groups. **(B)** The effects of AB treatment on relative levels of miR-155 between AG and CG groups.

Before the treatment, the difference for the levels of miR-146b and miR-155 was insignificant between two groups (Figures 3A,B, *P* > 0.05). After treatment, the expression of miR-146b and miR-155 in VMC children was significantly higher in the CG group than that of the AG group (Figures 3A,B, *P* < 0.05).

### Transcription factor expression levels

**Figure 4:**
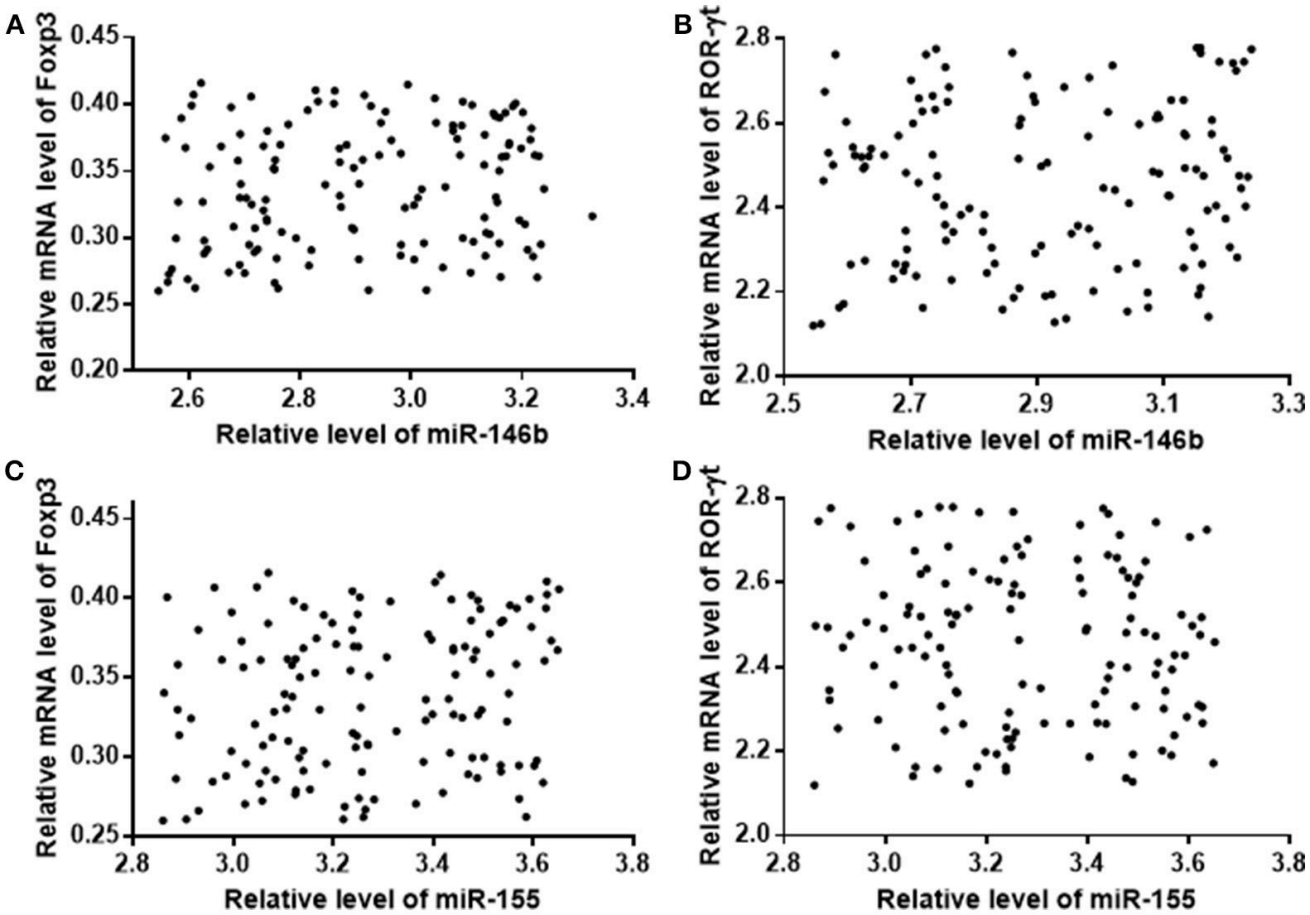
Pearson correlation coefficient analysis of the relationship between the expression levels of miR-146b and miR-155, and relative mRNA levels of FoxP3 and ROR-γt in Treg cells. **(A)** The relationship between the expression levels of miR-146b and relative mRNA levels of FoxP3. **(B)** The relationship between the expression levels of miR-146b and relative mRNA levels ROR-γt. **(C)** The relationship between the expression levels of miR-155 and relative mRNA levels of FoxP3. **(D)** The relationship between the expression levels of miR-155 and relative mRNA levels of ROR-γt. There is a strong negative relation between two variables if rho falls within from −0.5 to −1. There is a strong positive relation between two variables if rho falls within from 0.5 to 1. Relative mRNA levels of FoxP3 were negatively and the levels of ROR-γt were positively, associated with levels of miR-146b and miR-155.

Relative mRNA level of FoxP3 was lower in the CG group than in the AG group, whereas relative mRNA of ROR-γt was higher in the CG group than in the AG group (Table 5, *P* < 0.05). Pearson correlation analysis showed that the expression of miR-146b was negatively associated with FoxP3 level (Figure 4A, *P* < 0.05), and positively correlated with ROR-γt level (Figure 4B, *P* < 0.05). The expression of miR-155 was also negatively associated with FoxP3 level (Figure 4C, *P* < 0.05), and positively correlated with ROR-γt level (Figure 4D, *P* < 0.05).

**Table 5:** Comparison of the expression of peripheral blood transcription factor FoxP3 and ROR-γt.

	**Cases**	**FoxP3**	**ROR-γt**
VMC	68	0.34 ± 0.08	2.45 ± 0.33
Control	150	1.14 ± 0.15	0.93 ± 0.18
T		18.23	14.66
P		<0.05	<0.05

### The levels of cytokines in treg cells

**Figure 5:**
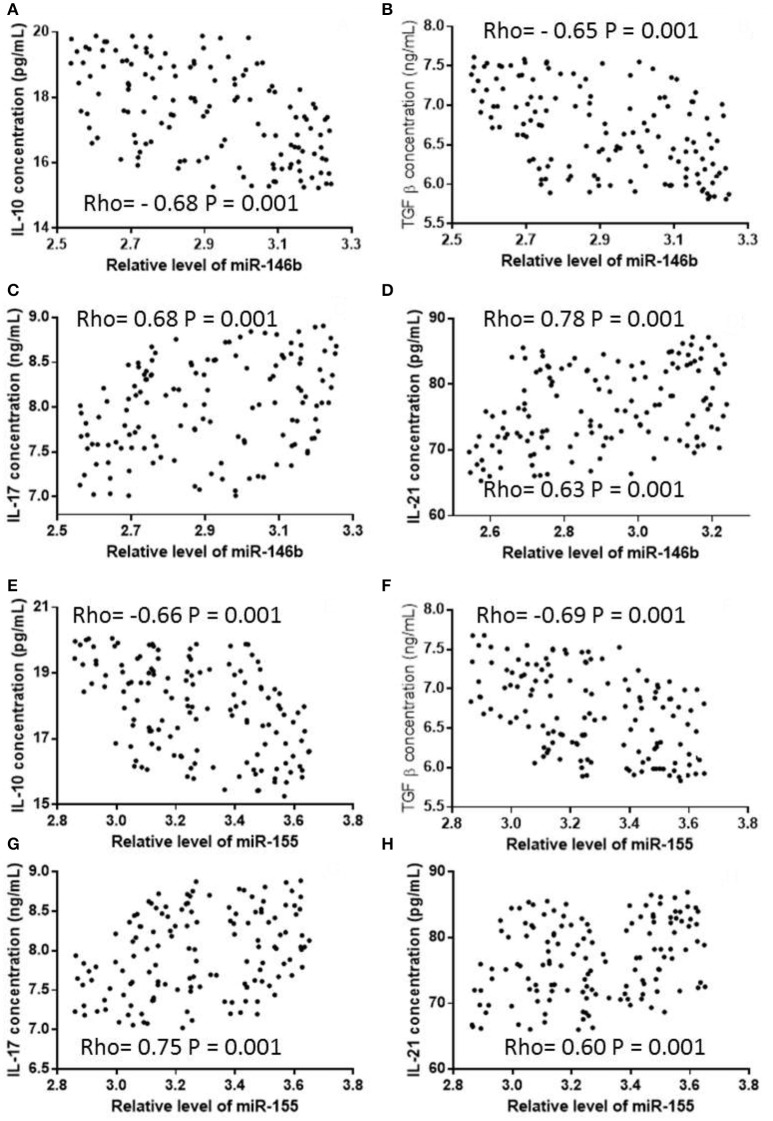
Pearson correlation coefficient analysis of the relationship between the expression levels of miR-146b and miR-155, and concentrations of cytokines in Treg cells. **(A)** The relationship between the expression levels of miR-146b and concentration of IL-10. **(B)** The relationship between the expression levels of miR-146b and concentration of TGF-β. **(C)** The relationship between the expression levels of miR-146b and concentration of IL-17. **(D)** The relationship between the expression levels of miR-146b and concentration of IL-21. **(E)** The relationship between the expression levels of miR-155 and concentration of IL-10. **(F)** The relationship between the expression levels of miR-155 and concentration of TGF-β. **(G)** The relationship between the expression levels of miR-155 and concentration of IL-17. **(H)** The relationship between the expression levels of miR-155 and concentration of IL-21. There is a strong negative relation between two variables if rho falls within from −0.5 to −1. There is a strong positive relation between two variables if rho falls within from 0.5 to 1. The levels of TGF-β and IL-10 were negatively and the levels of IL-17 and IL-20 were positively, associated with the levels of miR-146b and miR-155.

The levels of IL-10 and TGF-β in Treg cells were significantly lower in the CG group than those of the AG group, whereas the levels of IL-17 and IL-21 were significantly higher in the CG group than those in the AG group (Table 6, *P* < 0.05). Pearson correlation analysis showed that the expression of miR-146b was negatively associated with the levels of IL-10 (Figure 5A, *P* < 0.05) and TGF-β (Figure 5B, *P* < 0.05), and positively associated with the levels of IL-17 (Figure 5C, *P* < 0.05) and IL-21 (Figure 5D, *P* < 0.05). The expression of miR-155 was also negatively associated with the levels of IL-10 (Figure 5E, *P* < 0.05) and TGF-β (Figure 5F, *P* < 0.05), and positively associated with the levels of IL-17 (Figure 5G, *P* < 0.05) and IL-21 (Figure 5H, *P* < 0.05).

**Table 6:** Comparison of levels of IL-10, TGF-β, IL-17, and IL-21.

	**Cases, *n***	**IL-10 (pg/mL)**	**TGF-β (ng/mL)**	**IL-17 (ng/mL)**	**IL-21 (pg/mL)**
VMC	68	17.54 ± 2.35	6.74 ± 0.85	7.98 ± 0.94	75.90 ± 9.83
Control	150	32.48 ± 5.81	14.48 ± 1.80	3.38 ± 0.41	31.82 ± 4.71
T		8.57	12.38	13.46	11.54
P		<0.05	<0.05	<0.05	<0.05

### Myocardial injury indicators

**Figure 6:**
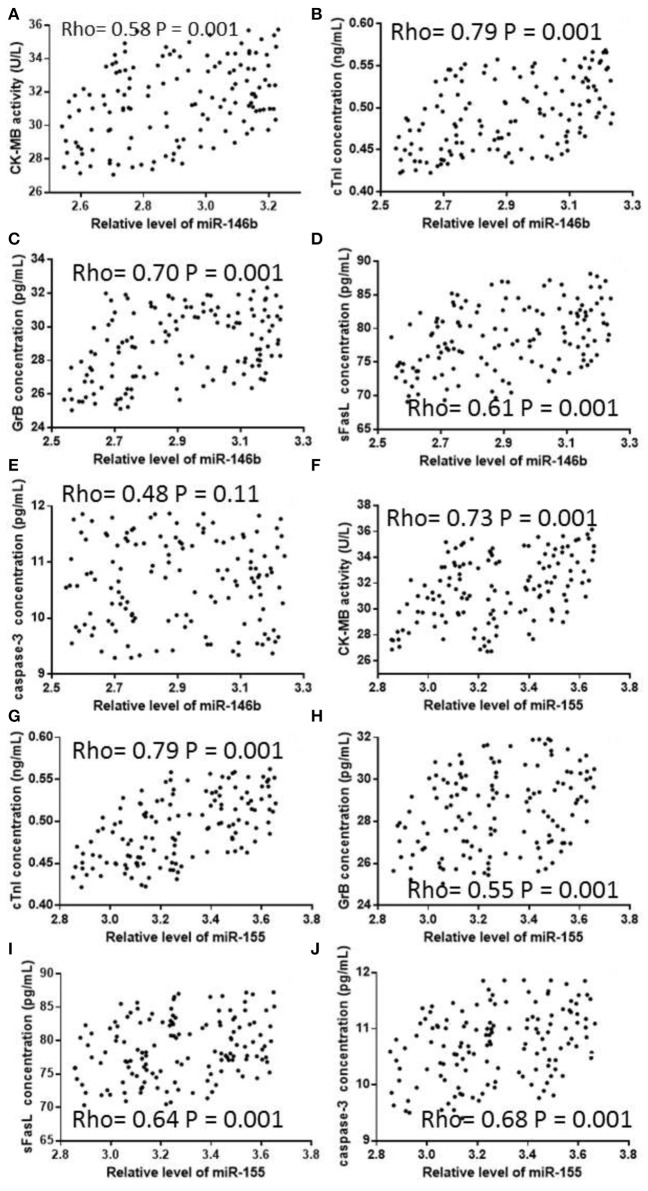
Pearson correlation coefficient analysis of the relationship between the expression levels of miR-146b and miR-155, and concentrations of the parameters related with myocarditis in Treg cells. **(A)** The relationship between the expression levels of miR-146b and concentration of CK-MB. **(B)** The relationship between the expression levels of miR-146b and concentration of cTnI. **(C)** The relationship between the expression levels of miR-146b and concentration of GrB. **(D)** The relationship between the expression levels of miR-146b and concentration of sFasL. **(E)** The relationship between the expression levels of miR-146b and concentration of caspase-3. **(F)** The relationship between the expression levels of miR-155 and concentration of CK-MB. **(G)** The relationship between the expression levels of miR-155 and concentration of cTnI. **(H)** The relationship between the expression levels of miR-155 and concentration of GrB. **(I)** The relationship between the expression levels of miR-155 and concentration of sFasL. **(J)** The relationship between the expression levels of miR-155 and concentration of caspase-3. There is a strong positive relation between two variables if rho falls within from 0.5 to 1. The levels of CK-MB, cTnI, GrB, and sFasL were positively associated with the levels of miR-146b and miR-155.

Levels of CK-MB, cTnI, GrB, sFasL and caspase-3 in children with VMC were significantly higher than those in the AG group (Table 7, *P* < 0.05). Pearson correlation analysis showed that the expression of miR-146b in Treg cells of children with VMC was positively associated with the contents of CK-MB (Figure 6A, *P* < 0.05), cTnI (Figure 6B, *P* < 0.05), GrB (Figure 6C, *P* < 0.05), sFasL (Figure 6D, *P* < 0.05) and caspase-3 (Figure 6E, *P* < 0.05). The expression of miR-146b in Treg cells of children with VMC was positively associated with the contents of CK-MB (Figure 6F, *P* < 0.05), cTnI (Figure 6G, *P* < 0.05), GrB (Figure 6H, *P* < 0.05), sFasL (Figure 6I, *P* < 0.05) and caspase-3 (Figure 6J, *P* < 0.05).

**Table 7:** Comparison of levels of the parameters related with myocardial injury.

	**Cases, *n***	**CK-MB (U/L)**	**cTnI (ng/mL)**	**GrB (pg/mL)**	**sFasL (pg/mL)**	**caspase-3 (pg/mL)**
VMC	68	31.24 ± 4.58	0.49 ± 0.07	28.51 ± 3.52	78.31 ± 9.02	10.59 ± 1.32
Control	150	10.36 ± 1.38	0.09 ± 0.02	11.24 ± 1.42	25.59 ± 3.12	2.58 ± 0.36
T		22.37	29.56	17.44	16.45	32.68
P		<0.05	<0.05	<0.05	<0.05	<0.05

### AB treatment increased the level of FoxP3 and reduced the level of ROR-γt in treg cells

**Figure 7:**
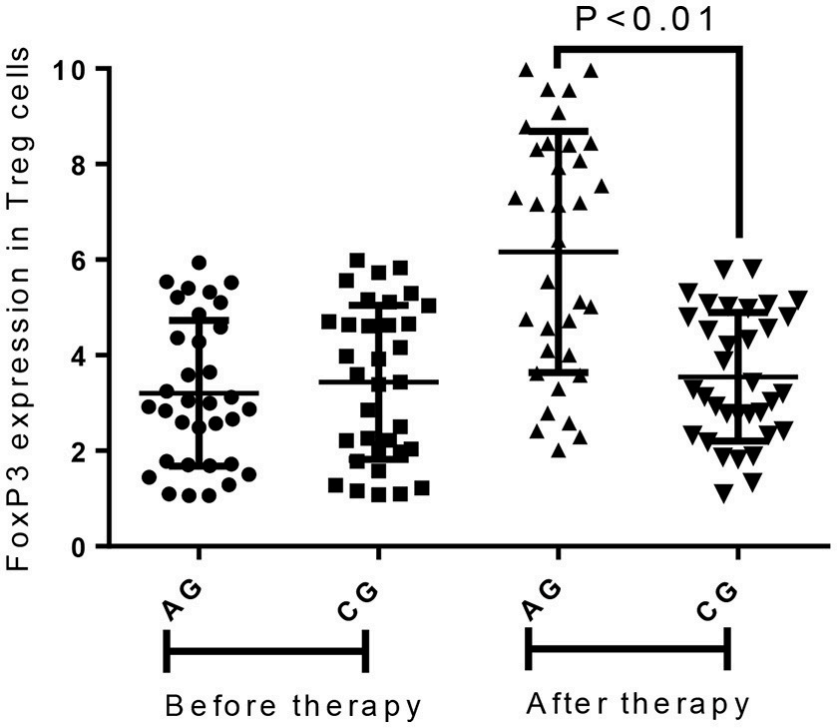
Flow cytometry analysis of Foxp3 expression in the Treg cells between different groups. All data were presented as the mean ± SD from three independent experiments.

**Figure 8:**
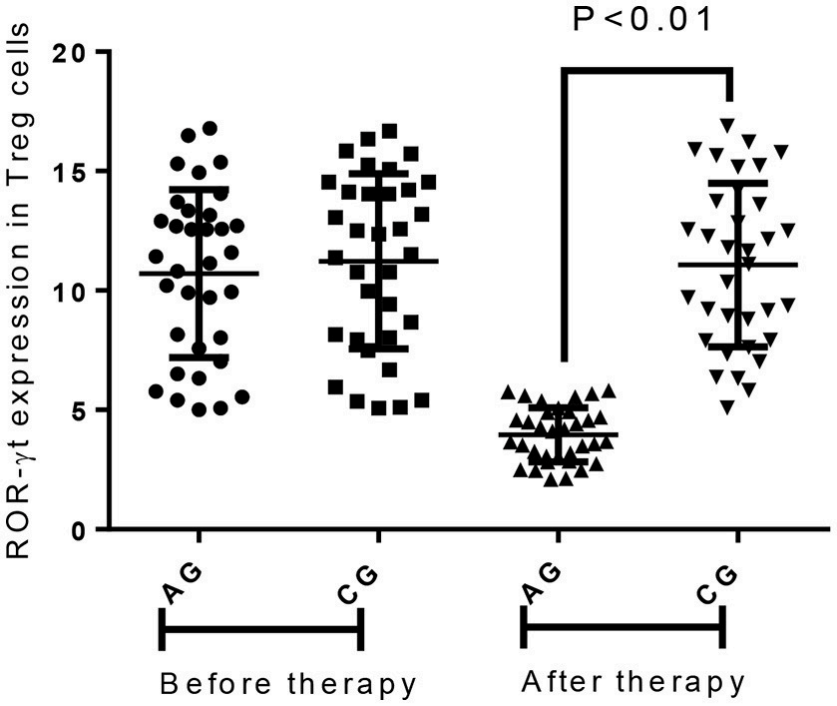
Flow cytometry analysis of RORγt expression in the Treg cells between different groups. All data were presented as the mean ± SD from three independent experiments.

Before the present experiment, the statistical difference was insignificant for the level of FoxP3 (Figure 7) and ROR-γt (Figure 8). Comparatively, AB treatment increased the level of FoxP3 (Figure 7) and reduced the level of ROR-γt (Figure 8). FoxP3 plays an important role for CD4^+^ CD25^+^ Treg function (27) whereas ROR-γt is the Th17-specific transcription factor (28). The results suggest that AB treatment may increase the relative contents of Treg cells and reduce the relative contents of Th-17 cells.

## Discussion

Astragalus contains a variety of flavonoids (29, 30) and polysaccharides compounds (31), which can not only effectively alleviate oxidant stress, but also improve the clinical manifestations of heart failure, and all of which are important to the prognosis of VMC. In this study, AB could effectively improve the fatigue, palpitations, chest pain and other clinical manifestations of VMC children when compared with those in the CG group. There were four main components (sucrose, calycosin, Astragaloside IV and calycosin-7-glucoside, Figure 1) in AB. Calycosin can effectively inhibit allergic inflammation and has anti-inflammatory properties (32). Astragaloside has been found to prevent myocardial cell apoptosis and protect myocardial cells, which is a kind of potential drug in the prevention of heart failure (33). Astragaloside IV has been found to have both antioxidant and anti-inflammatory activities (34). Anti-inflammatory, antioxidant can exert anti-tumor, antioxidant, and anti- hypertensive function (35). All these ingredients may contribute to the protective role of AB in VMC therapy.

miR-146b and miR-155 are two types of miRNAs with immunomodulatory effects. We analyzed the changes of the expression levels of the two kinds ofmiRNAs in the Treg cells of VMC children, and miR-146b and miR-155 expression was significantly higher in the CG group than the AG group. This showed that with the role of immune regulation of miR-146b and miR-155 increased expression and VMC was associated with the occurrence of myocardial damage caused by immune response. miR-146b and miR-155 abnormal expression may be involved in the development of VMC.

Treg cells are involved in important immune responses, which were regulated by miR-146b and miR-155 *in vivo*. Treg is an immune cell, and its differentiation and maturation is regulated by FoxP3, which can inhibit the activation of a variety of immune cells to suppress the immune response and reduce immune damage (36); ROR-γt regulates CD4^+^ T cell subsets via the secretion of IL-17 to cause tissue and cell damage (37). We analyzed the expression of the above two kinds of immune transcription factors in the Treg cells of VMC children, and found that the expression of FoxP3 in Treg cells of the CG group was significantly lower than that of the AG group, and the expression of ROR-γt was significantly higher than the AG group. Further analysis of miR-146b and miR-155 in Treg cells showed that miR-146b and miR-155 expression was negatively correlated with FoxP3 and ROR-γt (Figure 4).

The levels of IL-10 and TGF-β in the CG group were lower than those in the AG group, and the levels of IL-17 and IL-21 were higher in the CG group than the AG group. This shows that Treg cytokine secretion decreases Th17 cytokine secretion with VMC occurrence. Further analysis of miR-146b and miR-155 and Treg, Th17 cytokines correlation showed that miR-146b and miR-155 expression was negatively associated with IL-10, TGF-β content, and positively with IL-17 and IL-21 levels. This further confirmed that viral myocarditis in children with high expression of miR-146b and miR-155 on the secretion of Treg cytokines had an inhibitory effect on the secretion of Th17 cytokines promote role, and then causeed myocardial damage.

The levels of CK-MB, cTnI, GrB, sFasL, and caspase-3 in children with VMC were significantly higher in the CG group than those in the AG group. These results suggest AB treatment improves cardiac damage. Further analysis of the correlation between miR-146b and miR-155 in myocardial injury revealed that the levels of miR-146b and miR-155 in Treg cells of VMC children were significantly associated with the levels of CK-MB, cTnI, GrB, sFasL and caspase-3 (Figure 6). The results suggest that high expression of miR-146b and miR-155 can cause myocardial cell injury and apoptosis. The expression of miR-146b and miR-155 in Treg cells of VMC patients was highly expressed. High expression of miR-146b and miR-155 inhibited Treg immune response and promoted Th17 immune response, further aggravating cardiomyocyte injury and myocardial apoptosis. AB effectively reversed the process with few side effects.

## Conclusions

AB improves cardiac functions, peripheral Treg cell immunity imbalance in the children with VMC by reducing the levels of miR-146b and miR-155. The levels of miR-146b and miR-155 were higher in the CG group than in the AG group, suggesting that AB reduced the levels of miR-146b and miR-155. The levels of ROR-γt, FoxP3 and TGF-βwere lower in the CG group than in the AG group. In contrast, the levels of IL-17, IL-21, CK-MB, cTnI, GrB, sFasL and caspase-3 were higher in the CG group than in the AG group. Furthermore, the levels of ROR-γt, FoxP3, IL-10, and TGF-β were positively associated with the levels of miR-146b and miR-155, whereas the levels of IL-17, IL-21, CK-MB, cTnI, GrB, sFasL, and caspase-3 were negatively associated with correlated with the levels of miR-146b and miR-155. Therefore, evaluated levels of miR-146b and miR-155 were associated with Treg cell-mediated immunity imbalance in VMC children. The application of AB in the treatment of VMC children has potential clinical curative effect and few adverse reactions.

## Author contributions

ZZ and YL: conceived and designed the experiments, and wrote the paper; XD and JQ: performed the experiments; XD, YA, and CY: analyzed the data; YL: contributed reagents, materials, analysis tools.

### Conflict of interest statement

The authors declare that the research was conducted in the absence of any commercial or financial relationships that could be construed as a potential conflict of interest.
